# Constipation

**Published:** 1890-02

**Authors:** 


CONSTIPATION.—Much can often be done to overcome constipation by the free use of articles of food, such as cabbage, lettuce and the various vegetables known as greens; also, fruits, especially dates, prunes, figs, etc., and the coarser grains, cracked wheat, corn meal, articles with indigestible constituents, which stimulate or irritate the alimentary canal. A diet consisting, or made up in part, of the articles mentioned will materially assist in the cure of all cases, and sometimes entirely overcome habitual constipation. While food of this character is indicated in very many cases of constipation, there are yet some in which that disorder would be aggravated by it. Patients in whom digestion is weak, and there is a lack of tone to the intestines, are made more constipated by coarse vegetables and such fruits, etc. The same result follows the use of soft, doughy pastry, new bread and similar foods which are capable of being compressed into firm, hard masses, and which then resist the action of the digestive fluids.—*Boston Journal of Health.*

